# Bedside voluntary and evoked forces evaluation in intensive care unit patients, not only force evaluation: a letter to the Editor

**DOI:** 10.1186/s13054-021-03636-z

**Published:** 2021-07-01

**Authors:** Paulo Eugênio Silva, João Luiz Quaglioti Durigan, Nicolas Babault

**Affiliations:** 1 Research Group Fisiologia Clínica e Inovação Tecnológica (CNPq); Physical Therapy Division, Hospital de Base do Distrito Federal (IGESDF), Brasília, DF 70330-150 Brazil; 2 Graduate Program in Rehabilitation Sciences, Universidade de Brasília, Brasília, DF 70910-900 Brazil; 3 INSERM UMR1093 – CAPS, Faculty of Sport Sciences, University of Bourgogne Franche-Comté, 21000 Dijon, France

Dear Editor,

We read with great interest the article by Kennouche et al. [1] related to the evaluation of force in intensive care units (ICU). As indicated in their paper, numerous ICU patients develop a specific disease affecting their future quality of life; the so-called intensive care unit-acquired weakness (ICUAW). Early diagnosis associated with early treatment could limit these long-term effects but is challenging. Indeed, while some tools can be used in awake and cooperative individuals, an ICUAW diagnosis in sedated patients is technically difficult. For this reason, in this very well-conducted narrative review, the authors presented protocols that could be used to measure evoked force using electrical or magnetic stimulation in sedated individuals. In addition, tools and reliability data are presented.

Because ICUAW is often related to a reduction in force and muscle atrophy, the authors focused on the assessments of force. Nevertheless, beyond the scope of this review, electrical stimulation also allows the measurement of some neuromuscular electrophysiological disorders and should not be considered only for force evaluation. For example, an evaluation based on stimulus electrodiagnosis (i.e., motor excitability thresholds, for instance, with chronaxie data or H-reflex-Mwave recruitment curves) could be achieved with standard neuromuscular electrical stimulation [2]. Therefore, we believe this procedure should be used in complement with force evaluation. This is safe and feasible in the ICU and enables the detection of neuromuscular electrophysiological disorders [3].

As previously shown, force significantly decreases during the first days in the ICU. Early treatment with electrical stimulation is efficient to counteract these negative effects as it permits maintenance of force and avoids neuromuscular disorders [4]. However, early rehabilitation in the ICU is often complex for several technical reasons. As chronaxie rapidly increases over days in the ICU, we believe that electrodiagnosis could help prescribe optimized electrical stimulation treatments [4]. However, additional experiments should be conducted to determine the reliability of the different evaluation tools and protocols [1] and determine the best electrical stimulation rehabilitation procedure, characteristics, or modality (e.g., using belts or functional electrical stimulation) [5]. As indicated in this narrative review [1], electrical stimulation is an interesting tool for force evaluation in the ICU. However, electrical stimulation in the ICU should be considered in a more holistic manner as it represents a promising technique for improving patient health both during and after ICU stays.

## Data Availability

Not applicable.

## References

[1] Kennouche D, Luneau E, Lapole T, Morel J, Millet GY, Gondin J (2021). Bedside voluntary and evoked forces evaluation in intensive care unit patients: a narrative review. Crit Care.

[2] Lacomis D (2013). Electrophysiology of neuromuscular disorders in critical illness. Muscle Nerve.

[3] Silva PE, Babault N, Mazullo JB, Oliveira TP, Lemos BL, Carvalho VO (2017). Safety and feasibility of a neuromuscular electrical stimulation chronaxie-based protocol in critical ill patients: a prospective observational study. J Crit Care.

[4] Silva PE, De Cássia Marqueti R, Livino-De-Carvalho K, De Araujo AET, Castro J, Da Silva VM (2019). Neuromuscular electrical stimulation in critically ill traumatic brain injury patients attenuates muscle atrophy, neurophysiological disorders, and weakness: A randomized controlled trial. J Intensive Care.

[5] Al-Shekhlee A, Hachwi RN, Preston DC (2021). Early rehabilitation in ICU for COVID-19: what about FES-cycling?. Crit Care.

[6] Al-Shekhlee A, Hachwi RN, Preston DC, Katirji B (2005). New criteria for early electrodiagnosis of acute inflammatory demyelinating polyneuropathy. Muscle Nerve.

[7] Weber-Carstens S, Koch S, Spuler S, Spies CD, Bubser F, Wernecke KD (2009). Nonexcitable muscle membrane predicts intensive care unit-acquired paresis in mechanically ventilated, sedated patients. Crit Care Med.

[8] Grunow JJ, Goll M, Carbon NM, Liebl ME, Weber-Carstens S, Wollersheim T (2019). Differential contractile response of critically ill patients to neuromuscular electrical stimulation. Crit Care.

[9] Berney S, Hopkins RO, Rose JW, Koopman R, Puthucheary Z, Pastva A (2020). Functional electrical stimulation in-bed cycle ergometry in mechanically ventilated patients: a multicentre randomised controlled trial. Thorax.

