# Supplementary material for: Bedside voluntary and evoked forces evaluation in intensive care unit patients, not only force evaluation: a letter to the Editor
Source: Crit Care. 2021 Jul 1;25:233. doi: 10.1186/s13054-021-03636-z (PMC8252314; doi:10.1186/s13054-021-03636-z)
Supplement: Related texts

# Reply: ICUAW: Let’s focus on force evaluation

**Authors:** Djahid Kennouche, Eric Luneau, Thomas Lapole, Jérome Morel, Guillaume Y. Millet, Julien Gondin

**Affiliations:** 4 grid.25697.3f 0000 0001 2172 4233 Laboratoire Interuniversitaire de Biologie de la Motricité (LIBM), EA 7424, Univ Lyon, UJM-Saint-Etienne, Saint-Etienne, France; 5 grid.412954.f 0000 0004 1765 1491 Département d’anesthésie et de réanimation, Centre Hospitalier Universitaire, Saint-Etienne, France; 6 grid.440891.0 0000 0001 1931 4817 Institut Universitaire de France (IUF), Paris, France; 7 grid.7849.2 0000 0001 2150 7757 Institut NeuroMyoGène (INMG), Université Claude Bernard Lyon 1, CNRS UMR 5310, INSERM U1217, Lyon, France

Djahid Kennouche and Eric Luneau contributed equally to this work.

Guillaume Y. Millet and Julien Gondin contributed equally to this work.

We thank Silva and colleagues for their great emphasis and interest on our recent paper aiming at reviewing the different tools and methods allowing bedside force evaluation in ICU patients [1].

Although we appreciate the suggestion to add electrophysiological measurements in ICU patients, we believe this topic was beyond the scope of our review focusing on the impairment of muscle force during and after an ICU stay. We agree with Silva et al. that it can be an asset, as mentioned in the ‘Perspectives’ section: “*Evoked force measurements should also be combined with surface electromyography and ultrasound analyses […] to get a clear picture of the deleterious consequences of an ICU stay on the neuromuscular system…*”. Yet it should be kept in mind that (i) the use of chronaxie for ICUAW diagnosis has never been clinically validated; (ii) H-reflexes are usually absent in patients with critical illness polyneuropathy [6]; (iii) altered muscle membrane excitability [7] may prevent M-wave recordings (Kennouche et al., unpublished observations).

We also clearly highlighted the relevance of beside evoked force measurements for ICUAW treatment. So far, the application of neuromuscular electrical stimulation (NMES), a potential strategy for limiting muscle weakness and/or atrophy in ICU patients, is empirical and relies on a subjective quantification of muscle contraction intensity (*i.e.*, inspection of visible/palpable contractions) [8] as done for the chronaxie assessment. Bedside ergometers would therefore offer the unique opportunity to accurately quantify isometric contractile responses to NMES in ICU patients and could be used to provide evidence-based recommendations in future critical care NMES interventions.

Considering the difficulty in evoking contractile responses in all stimulated muscles and all patients [8], the possibility to produce coordinated movements by functional electrical stimulation (FES)-assisted cycling appears hypothetical in sedated patients. The effects of FES application on muscle strength of mechanically ventilated patients are also still equivocal [9].

As for NMES application, mechanical force/power output produced during FES cycling (e.g., using instrumented pedals) should be monitored to determine the effectiveness of FES on muscle force and mass of ICU patients. To conclude, bedside force measurements should remain at the heart of both diagnosis and treatment of ICUAW.
